# Effectiveness of modified Buzhong Yiqi decoction in treating myasthenia gravis: study protocol for a series of N-of-1 trials

**DOI:** 10.1186/s13063-022-06287-9

**Published:** 2022-04-27

**Authors:** Senhui Weng, Jinghao Li, Benshu Chen, Long He, Zhuotai Zhong, Linwen Huang, Shijing Zhang, Fengbin Liu, Qilong Jiang

**Affiliations:** 1 The First Clinical College, Guangzhou University of Chinese Medicine, Guangzhou, China; 2 Lingnan Medical Research Center of Guangzhou University of Chinese Medicine, Guangzhou, China; 3 The Second Clinical College, Guangzhou University of Chinese Medicine, Guangzhou, China; 4 School of Basic Medical Science, Guangzhou University of Chinese Medicine, Guangzhou, China; 5 Department of Gastroenterology, The First Affiliated Hospital of Guangzhou University of Chinese Medicine, Guangzhou, China; 6 Baiyun Hospital of the First Affiliated Hospital of Guangzhou University of Chinese Medicine, Guangzhou, China

**Keywords:** Modified Buzhong Yiqi decoction, Myasthenia gravis, N-of-1 trials, Traditional Chinese medicine, Gut microbiota

## Abstract

**Background:**

Myasthenia gravis (MG) is an acquired autoimmune disease with high heterogeneity. The disease is chronic, relapsing repeatedly and progressive with acute exacerbation occasionally. Although the treatment of MG has developed, it is still unsatisfactory and has some unexpected side effects. Traditional Chinese medicine (TCM) has shown great potential in MG treatment, including relief of muscle weakness syndrome, improvement of patient’s quality of life, and reduction of side effects of western medicine. The purpose of this study is to evaluate the effectiveness of modified Buzhong Yiqi decoction (MBYD) as an add-on therapy for MG through a small series of N-of-1 trials.

**Methods:**

Single-centre, randomized, double-blind, 3 crossover N-of-1 trials will be conducted to enroll patients with MG diagnosed as spleen-stomach deficiency syndrome or spleen-kidney deficiency syndrome in TCM. Each N-of-1 trial has 3 cycles of two 4-week periods containing the MBYD period and placebo period. The wash-out interval of 1 week is prior to switching each period. Primary outcome: quantitative myasthenia gravis (QMG). Secondary outcomes: the following scales: myasthenia gravis composite (MGC), myasthenia gravis activities of daily living profile (MG-ADL), myasthenia gravis quality of life (MG-QOL); the level of CD4+FoxP3+Treg cells and cytokines (IL-4, IL-17A, INF-γ, TGF-β) in the peripheral blood; the alterations of the composition of gut microbiota; reduction of the side effects of western medicine.

**Discussion:**

Used by WinBUGS software, we will conduct a hierarchical Bayesian statistical method to analyze the efficacy of MBYD in treating MG in individuals and populations. Some confounding variables such as TCM syndrome type and potential carryover effect of TCM will be introduced into the hierarchical Bayesian statistical method to improve the sensitivity and applicability of the trials, and the use of prior available information within the analysis may improve the sensitivity of the results of a series of N-of-1 trials, from both the individual and population level to study the efficacy of TCM syndrome differentiation. We assumed that this study would reveal that MBYD is effective for MG and provide robust evidence of the efficacy of TCM to treat MG.

**Trial registration:**

Chinese Clinical Trial Register, ID: ChiCTR2000040477, registration on 29 November 2020.

## Background

Myasthenia gravis (MG), characterized by weakness symptoms of various muscle groups, such as ptosis, dysphagia, dyspnea, limb weakness, and so on, is an antibody-mediated acquired autoimmune disease. The prevalence rate of MG ranges from 2.19 to 36.71 per 100,000, which depends on the geographic location [1]. Most MG patients (about 85%) have detectable antibodies against the acetylcholine receptor (AChR) in their serum [2]. It is a chronic, progressive, and stubborn neuromuscular disease that seriously affects MG patients’ quality of life.

The pathogenesis of MG has not entirely cleared. However, it was determined that it is highly correlated to immune dysregulation. CD4+T cells and their cytokines play a crucial role in the development and the progress of MG. CD4+ Foxp3+ regulatory T (Treg) cells, which are responsible for suppressing the immune response [3], have a protective pole in MG. Inflammatory cytokines such as interferon(IFN)-γ and interleukin(IL)-17 increase the anti-AChR level and aggravate MG weakness symptoms while anti-inflammatory cytokines such as transforming growth factor(TGF)-β and IL-4 downregulate anti-AChR level and alleviate weakness symptom [4, 5].

On the other hand, with the development of metagenomics, metabolomics, metatranscriptomics, and other omics, gut microbiota has attracted more and more attention and become an important frontier in understanding the development and progression of diseases [6]. In recent years, studies revealed that gut microbiota in MG patients has lower biodiversity and a lower *Firmicutes*/*Bacteroidetes* ratio as well as a significantly lower *Clostridium* content than that in healthy people. The disorder of gut microbiota promotes immune inflammation response, which aggravates the severity of MG [7, 8].

MG is usually treated with conventional western medicine such as acetylcholinesterase inhibitors, glucocorticoid, and other non-steroidal immunosuppressants with dissatisfactory clinical effects in some patients, the risk of unconscionable dosage, and unwanted adverse effects where traditional Chinese medicine (TCM) can take a complementary and alternative effect. A study found that using TCM combined with western medicine might improve the total effect and reduce the risk of MG relapse [9]. According to the TCM characteristic theory of “spleen governing muscle,” MG is generally diagnosed as “spleen *qi* deficiency syndrome” and thereby, strengthening the spleen and replenishing *qi* is a common therapeutic strategy of MG in TCM, which has played a positive role in clinical practice of MG [10, 11]. Buzhong Yiqi decoction (BYD), a classical *qi*-supplementing formula in TCM, is the most frequently used prescription to treat MG. A study showed that BYD modulated the balance of Treg and Th17 cells in patients with MG and downregulated the serum AChR antibody level [12]. Modified BYD (MBYD) is based on BYD and adds other Chinese herbs to treat MG patients’ specific or concurrent symptoms synergistically. A pharmacological study demonstrated that *Huangqi*, *Wuzhimaotao*, and *Gancao,* which are all in MBYD, have pharmacological components of acetylcholinesterase inhibitors [13]. Several clinical observations demonstrated that treating MG by combining MBYD with western medicine is more effective than western medicine alone [14–16]. However, high-level evidence of the effectiveness of MBYD for treating MG has yet to be constituted.

In this study, we will detect the level of Treg cells in the peripheral blood by flow cytometry and cytokines (IL-4, IL-17A, INF-γ, TGF-β) in serum by ELISA to investigate how MBYD modulates immunoregulatory cells and cytokines. What is more, studies have found that TCM contains complex compounds that, after taken orally, will inevitably interact with gut microbiota and modulate the structure and metabolism of gut microbiota and thereby obtain their therapeutic effects [17, 18]. We innovated to investigate the alterations of gut microbiota after TCM treatment in N-of-1 trials. This method has not been reported in previous studies. We collected stool samples from MG patients at baseline and after each cycle to determine the changes of the gut microbiota with MBYD treatment through multiple cross-controls.

### The rationales for the trials

Considering MG is a rare disease with high heterogeneity, conventional large-scale RCT trials are challenging to carry out. Single-case of the randomized controlled trial (also called “N-of-1”) is a multiple-crossover, two-phase cross design in a single patient to compare the efficacy of two interventions (or an intervention and a placebo), which requires an exceedingly smaller sample size than RCT [19]. There are some reasons why we will conduct this trial:

1. N-of-1 trials provide the most rigorous evidence possible of the effectiveness of the intervention in the individuals by multiple-crossover randomized controls [20]. Therefore, evidence from several N-of-1 trials can be gathered to create the efficacy estimates of the population treatment.
2. N-of-1 trials are fit for chronic, nonself-limited, relatively stable diseases requiring long-term medication treatment. Therefore, we set a rule that participants should have a stable phase in MG for at least 3 months.
3. TCM is characterized by syndrome differentiation and individualized intervention. N-of-1 trials can design various intervention measures flexibly for individuals. Hence, this method is relevant to the clinical practice of TCM. Nowadays, TCM N-of-1 trials, as the most compatible clinical method with TCM clinical diagnosis and treatment, have been widely concerned [21–23].

### Objectives

In this study, our objective is to comprehensively evaluate the effectiveness of MBYD in the treatment of MG. We assumed that this study would provide a credible conclusion that MBYD is an effective prescription for MG, which alleviates clinical symptoms, enhances the quality of life of MG patients, and reduces the side effects of western medicine. Furthermore, this study investigated the alterations of the level of Treg cells and cytokines (IL-4, IL-17A, TGF-β, IFN-γ) in peripheral blood and the gut microbiota in the intestinal tract of MG patients at baseline and after each phase so as to preliminarily explore how MBYD effects the immune system and gut microbiota in MG patients.

## Method

### Trial design

**Fig. 1:**
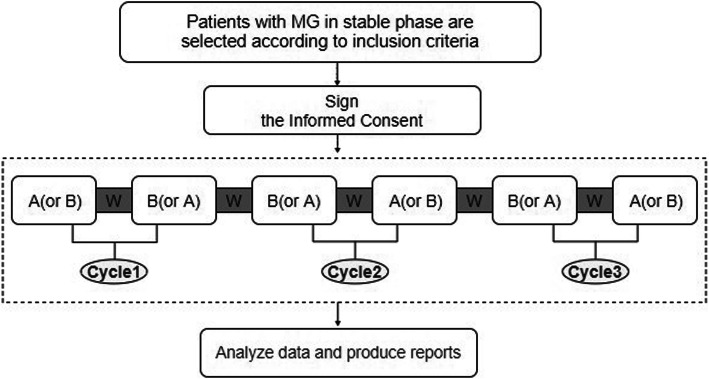
Design of the study. **A** is for the MBYD period. **B** is for the placeno period. **W** is for the wash-out interval. The order **A** and **B** in each cycle will be randomized. Figure not to scale

Because of the heterogeneous components of Chinese herbs, the onset time and half-time of Chinese herbs are hard to confirm [24]. According to previous clinical observation and preliminary trial [11], we decided to set the intervention period or control period for 4 weeks and the wash-out interval for 1 week. Each cycle comprises a 4-week intervention period with MBYD combining conventional western medicine and a 4-week control period with placebo with the same western medicine in random order. The wash-out interval prior to switching each period makes carryover effects minimized. Hence, there are 29 weeks in all involving 3 cycles in this study (Fig. 1). The conventional western medicine that the patients take still follows his/her original therapeutic schedule. In view that patients with MG cannot stop taking conventional western medicine for a few days, which may aggravate their condition rapidly and lead to serious adverse events, only MBYD or placebo granules are washed out while western medicine is maintained during the wash-out interval. The protocol is conducted in light of the SPIRIT reporting guidelines [25] and obeys the Conventional Protocol Items: CONSORT extension for reporting N-of-1 trials (CENT) 2015 and CONSORT extension for reporting N-of-1 trials for traditional Chinese medicine (CENT for TCM) 2019 [24, 26]. Table 1 shows the schedule of enrolment, interventions, and outcomes and safety assessments.

**Table 1:** Schedule of enrolment, interventions, outcomes, and safety assessments

Time point		Enrolment	Allocation	Cycle1		Cycle2		Cycle3		Close-out
				Weeks 1–4	Weeks 6–9	Weeks 11–14	Weeks 16–19	Weeks 21–24	Weeks 26–29	
Enrolment	Eligibility screen	√								
	Informed consent	√								
	Allocation		√							
Intervention	MBYD granules or placebo with the conventional western medicine			√	√	√	√	√	√	
Outcome	QMG score	√		√	√	√	√	√	√	√
	TCM syndrome score; MGC; MG-ADL; MG-QOL	√		√	√	√	√	√	√	√
	The level of CD4+ Foxp3+ Treg cells and cytokines (IL-4, IL-17A, INF-γ, TGF-β)	√		√	√	√	√	√	√	
	Gut microbiota	√		√	√	√	√	√	√	
Safety assessment	Laboratory tests	√		√	√	√	√	√	√	√
	Vital signs	√		√	√	√	√	√	√	√
	Adverse events	√		√	√	√	√	√	√	√

### Recruitment

The First Affiliated Hospital of Guangzhou University of Chinese Medicine (GZUCM) that we conduct the study is one of the centers for MG treatment in China and lots of MG patients will come to visit. Patients will be recruited as the participants from outpatient or resident physicians who professionally treat MG in GZUCM.

The patients will be selected if they adapt to the inclusion criteria as follows:

1. Patients aged between 18 and 65 and gender are not limited
2. Patients diagnosed with MG based on “The Chinese guidelines for the diagnosis and treatment of myasthenia gravis (2020)”: patients with typical clinical features of MG (fluctuating myasthenia) excluded from other diseases and meeting any of the following three points, including pharmacological examination, electrophysiological characteristics or serum antibody detection [27]
3. Patients attached to the spleen-stomach deficiency syndrome or spleen-kidney deficiency syndrome will be enrolled which is based on “The guidelines for clinical diagnosis and treatment of internal medicine of traditional Chinese Medicine: myasthenia gravis (2020)” [28]
4. Patients identified as class II or III according to myasthenia gravis foundation of America (MGFA) clinical classification [29] and are in the stable stage of MG at least 3 months
5. Patients with Quantitative Myasthenia Gravis (QMG) score more than 6
6. Patients treated with glucocorticoid administration should not take more than 15 mg prednisone (or an equivalent dose of other glucocorticoids) per day.

The exclusion criteria are following:

1. Female patients who are pregnant or lactating or have a pregnancy plan during the trial
2. Patients with other autoimmune diseases (e.g., polymyositis, multiple sclerosis, rheumatoid arthritis) that may impact the assessment and treatment
3. Patients with severe heart, kidney, liver, lung, hematological system, infectious diseases, or cancer
4. Patients with neuropsychiatric disorders that cannot cooperate
5. Patients received plasma exchange, glucocorticoid, or gamma-globin pulse therapy within 3 months
6. Patients received thymectomy within half a year
7. Patients with hypersensitivity of any drug in this trial
8. Patients participating in other trials
9. Patients consumed antibiotics, probiotics, or anti-acids during the previous 2 months

The withdrawal criteria are following:

1. Patients with poor adherence and not taking the trial medication as required by the study protocol
2. Patients who are allergic to the trial medication.
3. Patients with serious adverse effect (SAE) or suspected unexpected serious adverse reaction (SUSAR)
4. Patients with myasthenic crisis or worsening of symptoms requiring any treatment other than the trial medication
5. Patients who decide to withdraw from the trial or lose follow-up

The finished cycles before the withdrawal will be analyzed as part of the trial. We will still follow up with withdrawn participants until their planned end in order to appraise any adverse effects of the trial medication.

### Interventions

In consideration of the hardship in guaranteeing the quality of the decoction, we intend to use granules in this study. The conventional western medicine administration such as pyridostigmine, low-dose prednisone, and immunosuppressants is in the light of their prestudy treatment schedule. In order to avoid the effects of other Chinese herbs, any drug containing ingredients from Chinese herbs is not allowed except MBYD. Moreover, patients are not allowed to take antibiotics, probiotics, or anti-acids, which can obviously affect gut microbiota during the trial. For patients who develop an infection that requires antibiotics during the trials, they will suspend the cycle until symptoms resolve and antibiotics have been discontinued for 2 months, which is the time for the recovery of gut microbiota after antibiotics shown by the animal study [30]. After then, they will restart that cycle from the beginning.

The MBYD consists of *Astragalus Membranaceus* (*Huangqi*), *Radix Fici Simplicissimae* (*Wuzhimaotao*), *Rhizoma Atractylodis Macrocephalae* (*Baizhu*), *Radix Codonopsitis* (*Dangshen*)*, Poria Cocos* (*Fuling*), *Radix Bupleuri* (*Chaihu*), *Cimicifuga Foetida* (*Shengma*), *Pericarpium Citri Reticulatae* (*Chenpi*), *Angelica Sinensis* (*Danggui*), *Semen Coicis* (*Yiyiren*), *Rhizoma Dioscoreae* (*Shanyao*), *Radix Liquiritiae* (*Gancao*), and *Radix Scrophulariae* (*Xuanshen*). Patients with MG who have spleen *qi*-deficiency syndromes and kidney deficiency syndromes are usually diagnosed with spleen-kidney deficiency syndrome according to TCM theory and in that case, *Morinda officinalis* (*Bajitian*), *Cistanche Salsa* (*Roucongrong*), *Semen Cuscutae* (*Tusizi*), *Cornus Officinalis* (*Shanzhuyu*), *Taxillus sutchuenensis Danser* (*Sangjisheng*), and *Rosa laevigata Michx* (*Jinyingzi*), which are together used to replenish kidney *qi*, will be added in MBYD. Both the MBYD granules and the placebo granules were manufactured by Guangdong Yifang Pharmaceutical Co. Ltd.(10 g/bag, 2 bags three times a day, last number: J2004021). The placebo granules were identical to the MBYD granules in appearance, texture, color, and odor.

### Outcomes

Given the multi-component and multi-target characterization of TCM, an all-round assessment of its effective evaluation is significant. In this study, the primary outcome is the QMG score. The QMG score will be assessed by a physician with experience in MG according to the MGFA manual [29]. The QMG score has 13 items with 0–3 points for each item and a total score of 0–39 points. A higher QMG score indicates a more severe disease condition, and a decrease of 2.6 points is considered to be of clinical significance [31]. The secondary outcomes are the following:

1. TCM syndrome score. Assessing TCM syndrome score is based on “Diagnosis standards for common syndromes in traditional Chinese medicine” [32] issued by the Chinese Medicine Diagnostic Branch of China Association of Traditional Chinese Medicine. The measurement information of each symptom and sign of MG patient are weighted and summed according to the values of different symptoms and signs.
2. The scores of the following scales: myasthenia gravis composite (MGC), myasthenia gravis activities of daily living profile (MG-ADL), myasthenia gravis quality of life (MG-QOL)
3. The level of CD4+ Foxp3+ Treg cells and cytokines (IL-4, IL-17A, INF-γ, TGF-β) in the peripheral blood
4. The alteration of gut microbiota. Before the trials begin and after each period, we will collect the patients’ fresh fecal samples and then immediately store them in the laboratory freezer at − 80 °C until analyses. We will analyze the gut microbiota in the stool samples in order to explore the mechanism of MBYD in MG therapy at the microbiological level by the 16S rRNA gene sequencing.
5. The reduction and alleviation of side effects of western medicine for MG treatment

The safety assessments consist of the following items:

1. Laboratory tests, including serum alanine aminotransferase, aspartate aminotransferase, urea nitrogen, creatinine, and routine blood tests
2. Vital signs, physical examination, and electrocardiograph
3. Adverse events occurring during the study, including MG crisis, drug allergy, hepatorenal insufficiency, and other adverse drug reactions

The outcomes and the safety assessments will be conducted before the trials begin and at the end of each period.

### Sample size

We conducted the preliminary trials that 3 patients had finished all the three cycles. The mean and standard deviation (SD) of QMG scores (the primary outcome) difference between MBYD and placebo groups was (3.00±1.94). The sample size calculation was based on the effect of treatment with QMG scores [19]. We assumed that there were differences in treatment effect among different patients, and the difference value(Ψ) was 3.0. We used the two-sided test and set the type I error α of hypothesis test as 0.05 and the error β of class II as 0.2. Based on the estimate assuming 3 cycles, the sample size was calculated as 13 (random model) by using R 4.0 software [33–35]. Considering the 20% of lost follow-up rate, we set the sample size as 17.

### Randomization, blinding, and treatment allocation

We mark the intervention (MBYD) as A and the control (placebo) as B. Then, each cycle can be recorded as AB or BA. The clinical research associate (REA) uses SPSS 26.0 to set a random seed and generate adequate random numbers in the range of (0,10) intervals, and all the random numbers will be rounded down. Each generated odd number is regarded as AB and an even number is regarded as BA. The first to third digits are allotted to the first participant, the fourth to sixth digits are allotted to the second participant, and so on. For example, if a series of random numbers generated by a computer is “453728…”, then the first three numbers “453” are given to the first participant and thus the corresponding order of the trial for him/her is “BA-AB-AB” and homoplastically, the next three numbers “728” are given to the second participant and the corresponding order of the trial for him/her is “AB-BA-BA”, and so on.

The allocation result will be written and put into an opaque and sealed envelope that will not be opened until the end of the trials. The granule storage and distribution will be in charge of the REA. Participants and investigators will both be blinded until the end of the trials.

In our experience, MBYD is usually mild and fewer side effects. In the case of SAEs related to MBYD, the supervisor and primary researchers shall decide whether emergency blind breaking is necessary, in order to know what kind of medicine the patient is taking in time. When breaking the blind, researchers should write the date, the reason for opening the blind, and sign on the envelope.

### Data management and monitoring

In the trials, researchers will record and collect all data on the CRFs. The collecting data will be input using EpiData 3.0(EpiData Association, Odense, Denmark). To guarantee the accuracy and completeness of the data, double data input and proofreading will be carried out by two independent researchers. The individual data of the participants and the data generated during the study will be kept confidential by one researcher, and the result report after the study will not reveal the personal identity of the participants. the State Food and Drug Administration, the Ethics Committee, investigator, and sponsor representatives will be allowed access to participant data in order to verify clinical study procedures and/or data. We will do everything within existing laws to protect the privacy of our participants. A data monitoring plan will be formulated and the study data will be monitored by independent data monitors. All adverse events of the participants will be recorded in detail, properly handled, and tracked until the conditions are properly resolved or stabilized, and SAEs and unexpected events will be reported to the Ethics Committee, competent authorities, sponsors and drug regulatory authorities in a timely manner as required; The primary residents periodically conducted cumulative reviews of all adverse events, and convened investigator meetings when necessary to assess the risks and benefits of the study.

### Follow-up after the trial

Patients who have finished all the cycles will be invited to the research center to follow up after 1 month, researchers will evaluate QMG, TCM syndrome scale, MGC,MG-QOL,MG-ADL and the safety assessments. If the patient’s result report reveals MBYD is beneficial to treat MG, we would give him/her a suggestion that MBYD can be used as an adjunct medicine with the conventional western medicine to alleviate muscle weakness symptoms and enhance the quality of life.

### Statistical methods

MG is a rare disease with high heterogeneity among patients. Bayesian analysis methods will be used to analyze the efficacy of MBYD in treating MG in individuals and populations. Hierarchical Bayesian statistical method is on account of the Monte Carlo technology of Markov’s Bayesian hierarchical model calculation chain, using WinBUGS software for calculation. To combine the results of the multiple N-of-1 trials, a hierarchical model will be used to analyze the difference in efficacy under stratification. The confounding variables such as TCM syndrome differentiation and the potential carryover effect of TCM will be used as the structural grouping factors (or the levels of the model). Suppose there is a common internal residual in the trials. For this multistage model, the trials will use a Bayesian approach to combine data from individual N-of-1 trials. We have elicited a prior distribution for the mean difference effect: a normal distribution with mean 3.00 and a SD of 1.94 [36]. Hence, we will acquire marginal posterior distribution for the mean treatment effects at the population levels and for the variations between patients, as well as a posterior distribution of treatment effects at the individual level, which will exhibit borrowed power through contraction from the population estimates to the population mean. For more details about the program used, see Zucker et al. (2006, especially the appendix) [37]. The probability distributions of each model will be analyzed to assess violations and data transformations undertaken, where necessary. Conventional burn-in periods, model convergence and stability diagnostics as well as residual checks will be adopted. The patterns of hierarchical Bayesian statistics analysis results will be as follows: (1) the mean of the posterior distribution of the mean difference between the outcomes of MBYD and placebo, which provides the best evaluation of the difference in the overall effect size between the treatments; (2) the relevant 95% credible region, which gives uncertainty interval of the posterior distribution (2.5 and 97.5 percentiles in this case), and (3) the posterior probability of mean difference that MBYD will be better than the placebo, which describes the likelihood that the patients will squint towards actively treat in future cycles. At the time that these estimated values are better than predetermined threshold values (e.g., the increment of the average of QMG score is ≥3 points), the patient will be defined as a “responder” [33, 38, 39].

### Auditing

A research assistant supervises the sequence and procedure of the trial, and this process is independent from the other investigators.

## Discussion

According to our rich clinical experience, we found that TCM treatment can alleviate fatigue and muscle weakness and improve the quality of life of MG patients. A series of N-of-1 trials is currently a helpful tool for maximizing clinical benefits for an individual patient and has significant potential to supply convincing effective information for most clinical fields [40]. It can be seen as conducting multiple RCTs on individuals. TCM is highly consistent with N-of-1 trials because TCM is characterized by syndrome differentiation and holism emphasizing individualized treatment. In recent years, more and more scholars have paid attention to TCM N-of-1 trials.

It is challenging to assess the effectiveness of TCM because of its heterogeneous ingredients, multiple targets, and miscellaneous effects. In this study, we plan to use QMG, MGC, and MG-ADL clinical rating scales to evaluate the severity of MG and MG-QOL for assessing the quality of life in MG patients and the TCM symptom scale for analyzing the syndrome improvement. Meanwhile, the level of Treg cells and related cytokines are also our outcomes so as to comprehensively evaluate the effectiveness of TCM. In addition, the changes of gut microbiota through TCM treatment can also be used as a method to evaluate the effectiveness of TCM. Therefore, we innovated to use multiple crossovers within the N-of-1 trials for detecting and analyzing the composition of gut microbiota, aiming to form credible evidence that the effect of TCM on how to modulate the composition of gut microbiota in MG patients.

For TCM N-of-1 trials, previous studies published or conducted have an inferior reporting quality [41]. On the one hand, because of the multi-target and confounding effect of TCM, it is hard to determine the half-time and onset time, which brings the difficulty to set the period of intervention and wash-out. On the other hand, how to evaluate the clinical effectiveness of TCM still needs to be further improved. Besides, a part of TCM treatments may have a slow onset of clinical effects and do not show curative effects in a short period, which brings some difficulties to the conductions of TCM N-of-1 trials. Nevertheless, TCM N-of-1 trials still have great potential and their future is worth looking forward to.

## Trial status

The recruitment began on 5 Dec 2020, and we expect this study to be completed by 30 May 2022.

## Abbreviations

MG: Myasthenia gravis
QMG: Quantitative myasthenia gravis
TCM: Traditional Chinese medicine
MGC: Myasthenia gravis composite
MG-ADL: Myasthenia gravis activities of daily living profile
MG-QOL: Myasthenia gravis quality of life
BYD: Buzhong Yiqi decoction
MBYD: Modified Buzhong Yiqi decoction
N-of-1: Single-case randomized controlled trial
RCT: Randomized controlled trial
GZUCM: Guangzhou University of Chinese Medicine
CENT: CONSORT extension for reporting N-of-1 trials
CRF: Case report form
AChR: Acetylcholine receptor
Treg cell: Regulatory T cell
INF-γ: Interferon-γ
TGF-β: Transforming growth factor-β
IL: Interleukin
SAE: Serious adverse effect
SD: Standard deviation
REA: Research associate
